# Pheromones modulate reward responsiveness and non-associative learning in honey bees

**DOI:** 10.1038/s41598-017-10113-7

**Published:** 2017-08-29

**Authors:** David Baracchi, Jean-Marc Devaud, Patrizia d’Ettorre, Martin Giurfa

**Affiliations:** 1 Research Centre on Animal Cognition, Center for Integrative Biology, CNRS, University of Toulouse, 118 route de Narbonne, F-31062 Toulouse Cedex 09, France; 2 Laboratory of Experimental and Comparative Ethology, University of Paris 13, Sorbonne Paris Cité, France

## Abstract

Pheromones are chemical messengers that trigger stereotyped behaviors and/or physiological processes in individuals of the same species. Recent reports suggest that pheromones can modulate behaviors not directly related to the pheromonal message itself and contribute, in this way, to behavioral plasticity. We tested this hypothesis by studying the effect of pheromones on sucrose responsiveness and habituation in honey bees. We exposed workers to three pheromone components: geraniol, which in nature is used in an appetitive context, and isopentyl acetate (IPA) and 2-heptanone (2H), which signal aversive situations. Pheromones associated with an aversive context induced a significant decrease of sucrose responsiveness as 40% and 60% of bees exposed to IPA and 2H, respectively, did not respond to any sucrose concentration. In bees that responded to sucrose, geraniol enhanced sucrose responsiveness while 2H, but not IPA, had the opposite effect. Geraniol and IPA had no effect on habituation while 2H induced faster habituation than controls. Overall, our results demonstrate that pheromones modulate reward responsiveness and to a lower degree habituation. Through their effect on sucrose responsiveness they could also affect appetitive associative learning. Thus, besides conveying stereotyped messages, pheromones may contribute to individual and colony-level plasticity by modulating motivational state and learning performances.

## Introduction

Pheromones are chemical substances released to the environment by an individual, which convey specific messages and trigger stereotyped behaviors or physiological processes in individuals of the same species^1^. Pheromones are, therefore, fundamental key-players in animal communication and mediate a variety of responses in a broad spectrum of behavioral and ecological contexts. Pheromone-elicited responses are typically predictable and innate in the sense that they do not require specific learning^2^. Yet, in recent years, a novel twist in the consideration of pheromone actions has been proposed. Precisely, pheromones have been suggested as modulators of a variety of responses that are not necessarily related to the pheromonal message itself. In particular, pheromones have been shown to influence cognitive tasks, thereby affecting the capacity of an animal to learn and memorize specific information. For instance, exposure to a putative stress-related anxiogenic pheromone released by a stressed mouse impairs aversive conditioning of a conspecific receiver^3^. Similarly, exposure of young worker honey bees to the queen pheromone blocks their capacity to learn aversive associations while leaving intact their capacity to learn appetitive associations^4^. These two examples show that in some cases pheromones act on behaviors that are not the primary target of their action, affecting their intensity, success or probability of occurrence. As such, they may act as modulators of behavioral plasticity.

Here, we aimed at studying the rules and mechanisms of pheromonal modulation of behavior in the honey bee, an insect which constitutes one of the pinnacles of social organization among animals^5^. The social life style of honey bees, with their highly efficient division of labor^6–8^, relies to a high extent on pheromones that regulate multiple social interactions and individual behaviors^9^. Several pheromones have been identified in this insect and their role as releasers or primers of different behaviors has been thoroughly characterized^9^. Moreover, neural circuits of pheromone processing have also been studied in bees^10–13^, thus making this insect an appropriate model for studying the impact of pheromones on behaviors not strictly related to the pheromonal message considered. Here we focused on three bee pheromone components that differ in valence and social context: geraniol, 2-heptanone (2H) and isopentyl acetate (IPA). Geraniol is the main component of the Nasanov gland, which elicits attraction and aggregation of receiver workers^14^. As this pheromone component signals valuable resources, triggers attraction and relates to appetitive searching behavior motivation, we refer to it henceforth as “positive-valence pheromone”. The single-component pheromone 2-heptanone (2H) is an alarm substance released by the mandibular glands, which exerts a repellent action on intruders and robbers from other hives^15, 16^, but which has been also suggested as a deterrent signal during foraging to mark visited and depleted flowers^17–19^. Isopentyl acetate (IPA) is the main component of the sting alarm pheromone released by the Koschevnikov gland, which causes receiver bees to sting, attack^15, 20^ and stop foraging^21–23^. As 2H and IPA signal situations or stimuli to be attacked or avoided as dangerous, potentially noxious and/or negatively-valued, we refer to them as “negative-valence pheromones”.

We studied the impact of these pheromone components on two different behaviors: the subjective evaluation of sucrose reward, which may be assessed via the innate responsiveness of bees to sucrose solutions of increasing concentration, and habituation to antennal sucrose stimulation, which is a case of non-associative learning. Both behaviors are quantified via the proboscis extension reflex (PER), which is the appetitive response of bees to sucrose reward perceived via the antennae. Sucrose responsiveness, on the one hand, has received particular attention in the framework of studies on division of labor and social organization in bees^24–27^. Indeed, bees within a colony differ in their sucrose responsiveness, a fact that translates into the fine specializations existing within the forager caste and thus into the decision to collect nectar, pollen or water^26–28^. Habituation, on the other hand, is the progressive and reversible decrease of responsiveness to a significant stimulus that is delivered repeatedly and predictably to an animal^29, 30^. Focus on habituation is justified given the correlation existing between sucrose responsiveness and habituation to antennal sucrose stimulation^31^: bees with high responsiveness to sucrose display a lower degree of habituation and show greater dishabituation than bees with low responsiveness. Here we studied the modulatory effect of pheromone components on sucrose responsiveness and habituation as potential changes in these behaviors were quantified at least 15 min after pheromone exposure, when the substances were no longer present. We excluded in this way reflexive responses and acute effects of pheromone components.

We hypothesize that positive- and negative-valence pheromones exert different modulatory effects on these behaviors: while the former would increase sucrose responsiveness and decrease habituation, the latter would induce opposite effects. According to this view, pheromones (and their main components) would modulate the bees’ subjective evaluation of reward and their motivation to learn about appetitive situations.

## Results

### Effect of pheromone exposure on sucrose-reward responsiveness

We first evaluated the capacity of geraniol, 2H and IPA to modulate sucrose responsiveness in forager honey bees. To this end, we determined whether pheromone exposure changes the responses of worker bees to successive stimulations with six increasing sucrose concentrations (from 0.1/% to 30% w/w)^28^. Bees were exposed either to geraniol, IPA or 2H. Control bees were exposed to mineral oil (solvent). PER to each sucrose stimulation was recorded and responses were quantified in terms of a sucrose responsiveness score (SRS)^25^. SRS is defined as the number of sucrose concentrations to which a bee actually responded (i.e. a SRS of 6 corresponds to a bee that responded to all six concentrations, while a SRS of 1 corresponds to a bee that only responded to the highest sucrose concentration)^25^.

**Figure 1:**
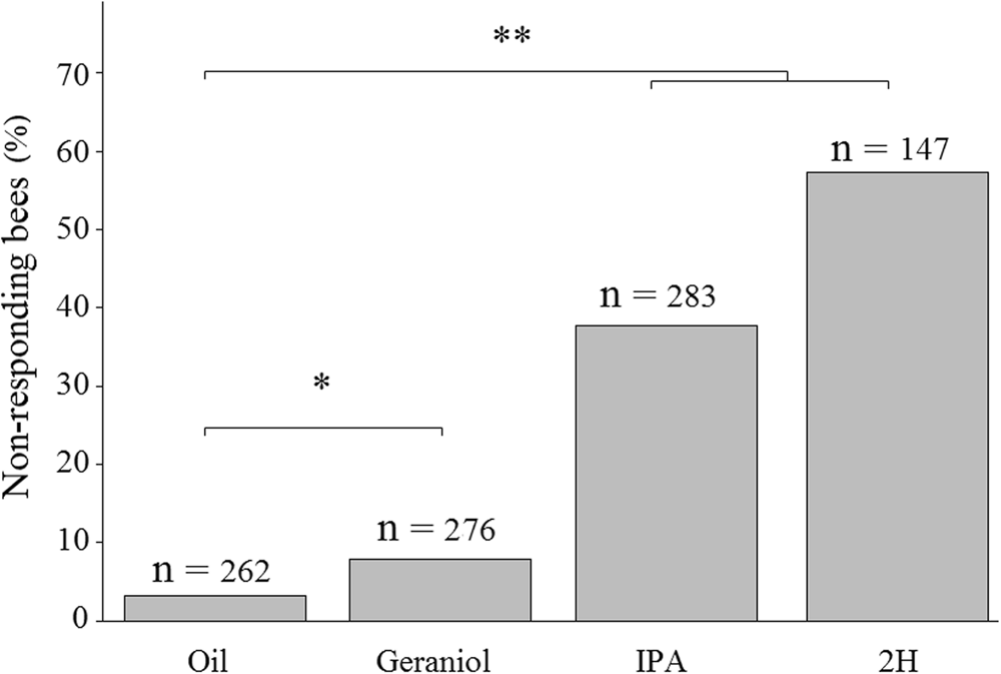
Pheromones affect sucrose responsiveness. Proportions of bees that failed to respond to any of the tested sucrose concentrations (including an additional sucrose concentration of 50% w/w delivered at the end of the stimulation sequence) (*non-responding bees*) following exposure to mineral oil or to one of the three pheromone components (geraniol, IPA and 2H). *p < 0.01; **p < 0.0001.

After pheromone exposure, significantly more bees failed to respond to any sucrose concentration when compared to control bees. Only 3.1% of the control bees exposed to mineral oil did not respond to any sucrose concentration, including an additional higher concentration of 50% (w/w). For bees exposed to geraniol, the proportion of these non-responders increased to 8.6%, while it was 38.5% and 58.5% for bees exposed to IPA and 2H, respectively (*geraniol vs control:* χ^2^ = 7.6, df = 1, p = 0.01; *IPA vs control*: χ^2^ = 101.5, df = 1, p < 0.001; *2H* *vs control*: χ^2^ = 163.6, df = 1, p < 0.001) (Fig. 1).

**Figure 2:**
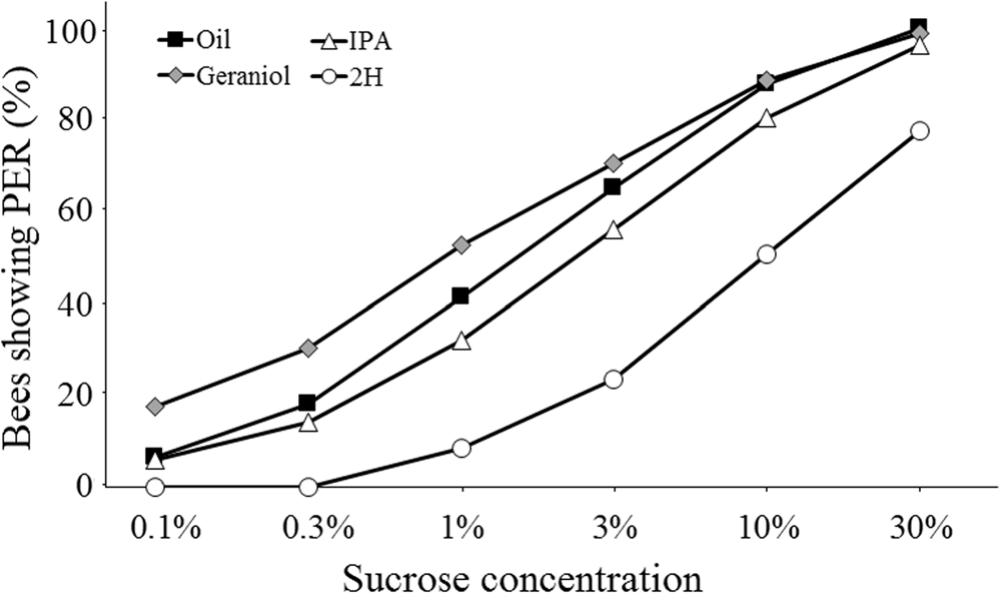
Pheromone exposure affects sucrose responsiveness. Cumulative proportions of bees showing PER when presented with the six sucrose solutions of increasing concentration (0.1%, 0.3%, 1%, 3%, 10% and 30% w/w). Bees exposed to geraniol (n = 252) and 2H (n = 61) were respectively more and less responsive than control bees exposed to mineral oil (n = 254), (GLMM, *geraniol vs oil:* p = 0.002; *2H* *vs oil:* p < 0.001). IPA (n = 174) had no significant effect on sucrose responsiveness (*IPA vs oil:* p = 0.46).

Bees responding at least to the 50% sucrose concentration were used to compare sucrose responsiveness among groups and to establish individual sucrose response scores (SRS). The responses of bees exposed to different pheromone components or mineral oil differed between treatments and sucrose concentrations (GLMM, *pheromone*: χ^2^ = 70.25, df = 3, p < 0.001; *sucrose concentration:* χ^2^ = 254.40, df = 5, p < 0.001) (Fig. 2). As expected, bees responded more to sucrose solution of increasing concentrations, but this increase was enhanced in bees exposed to geraniol and reduced in those exposed to 2H when compared to control bees (*geraniol*: n = 252, p = 0.002; *2H* *:* n = 61, p < 0.001). IPA exposure did not affect sucrose responsiveness (n = 174, p = 0.46).

**Figure 3:**
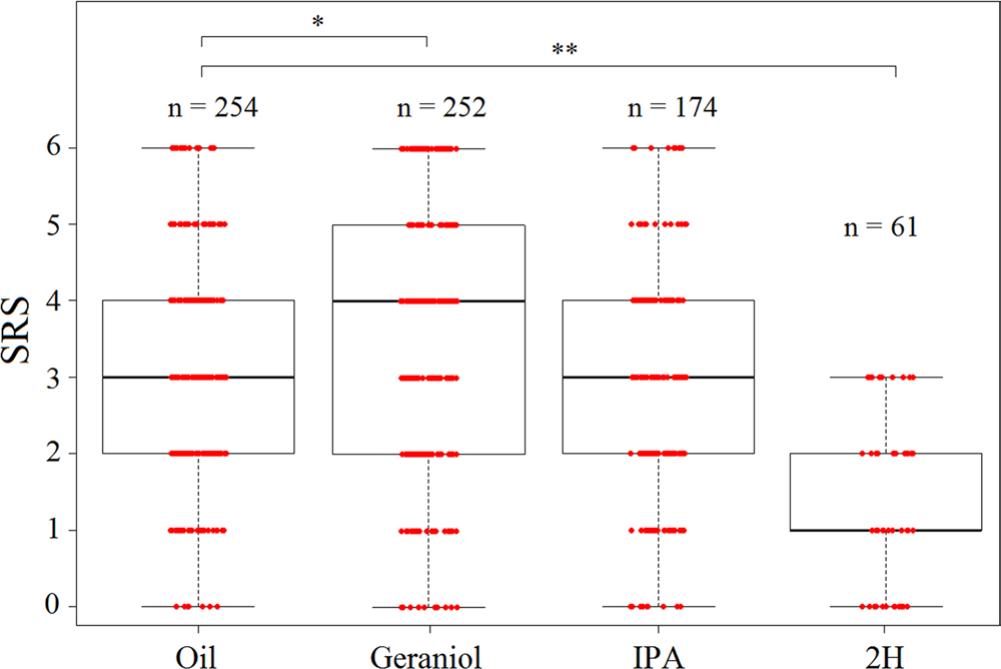
Pheromone exposure affects individual sucrose response scores (SRS). Median, quartiles and max and min (upper and lower whiskers) SRS values of bees exposed to either mineral oil or to one of the three pheromone components (geraniol, IPA and 2H). Red dots represent individual bees. For each bee, SRS was established by measuring PER to a series of six sucrose solutions of increasing concentration (0.1%, 0.3%, 1%, 3%, 10%, and 30% w/w). SRS values ranged between six (bees responding to all 6 concentrations) and 0 (bees not responding to any concentration). *Non-responding bees* (i.e. bees not responding even to an additional concentration of 50%; see Fig. 1) were excluded from this analysis as their SRS value could not be established. *p = 0.045; **p < 0.0001.

Accordingly, the SRS, which provides an individual assessment of sucrose responsiveness, varied significantly across groups, i.e. with pheromone exposure (Kruskal-Wallis test, H = 70.0, df = 3, p < 0.001) (Fig. 3). Compared to control bees, geraniol-exposed bees had significantly higher SRS (Mann-Whitney U-test, U = 27739, p = 0.048). Notably, a higher proportion of geraniol-exposed bees responded to intermediate concentrations of sucrose (0.1%: χ^2^ = 14.2, p = 0.001; 0.3%: χ^2^ = 9.5, p = 0.01) (Fig. 2). On the contrary, 2H strongly decreased SRS, thus making bees less responsive to sucrose (U = 3449, p < 0.001). In particular, none of the 2H-exposed bees responded to the lowest sucrose concentrations of 0.1% or 0.3%, and responses to the higher concentrations were always below those of control bees (p < 0.01, in all cases) (Fig. 2). By contrast, IPA had no significant effect on SRS (U = 24872, p = 0.15) (Fig. 3).

**Figure 4:**
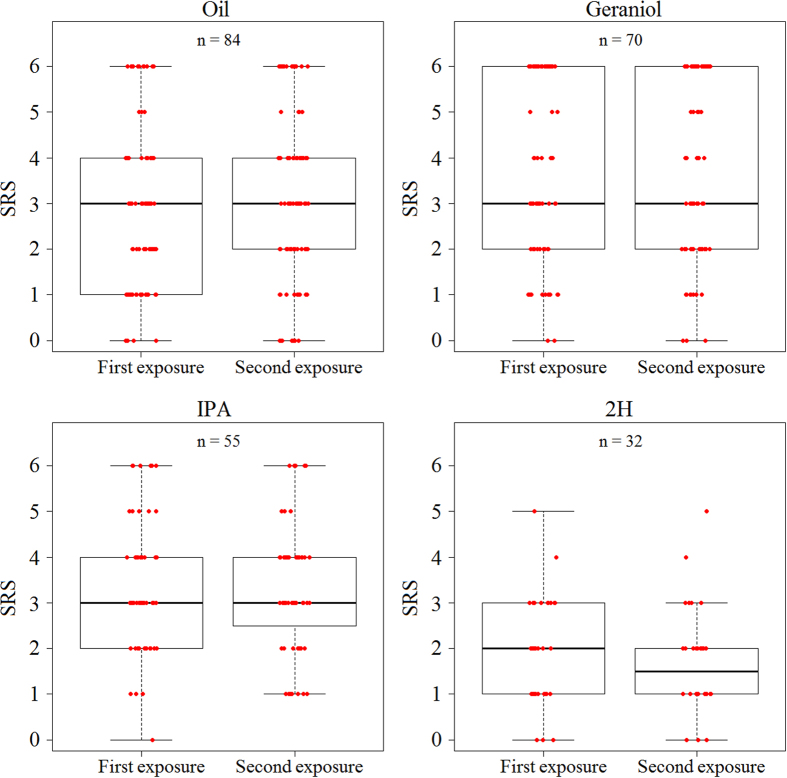
Sucrose response scores are similar following repeated exposures. Median, quartiles and max and min (upper and lower whiskers) SRS values of bees evaluated after a first and second exposure to mineral oil or to one of the three pheromone components (geraniol, IPA and 2H). Red dots represent individual bees. For each bee, SRS was established by measuring PER to a series of six sucrose solutions of increasing concentration (from 0.1% to 30%). SRS values ranged between six (bees responding to all 6 concentrations) and 0 (bees not responding to any concentration). For each of the two sessions, *non-responding bees* (i.e. bees not responding even to an additional concentration of 50%) were discarded as their SRS value could not be established. In all groups, the SRS remained invariable between the two successive exposures (Wilcoxon test, *oil*: n = 84, p = 0.18; *geraniol*: n = 70, p = 0.96; *IPA*: n = 55, p = 0.98. *2H*: n = 32, p = 0.27).

We then asked whether the modulatory effects of pheromones on sucrose responsiveness were consistent over successive exposures to the same pheromone. To answer this question, bees were exposed twice to a given pheromone component and sucrose responsiveness was quantified after each exposure. Two hours elapsed between the two pheromone exposures. A correlation analysis showed that control bees exposed twice to mineral oil showed consistent SRS values (Spearman corr. test, n = 84, ρ = 0.66, p < 0.001). Similarly, SRS after two successive pheromone exposures were highly correlated: both geraniol exposures determined a similar increase of sucrose responsiveness (Spearman: n = 70, ρ = 0.8, p < 0.001) while the two 2H exposures induced a similar decrease of sucrose responsiveness (n = 32, ρ = 0.4, p < 0.001). In the case of IPA, both exposures did not change sucrose responsiveness (n = 55, ρ = 0.4, p < 0.01). A comparison of SRS between both exposures (Fig. 4) confirmed that responsiveness remained constant within each treatment (Wilcoxon test, *oil*: n = 84, W = 3074, p = 0.18; *geraniol*: n = 70, W = 2439, p = 0.96; *IPA*: n = 55, W = 1517, p = 0.98; *2H*: n = 32, W = 591, p = 0.27). Moreover, the proportion of bees that did not respond to any sucrose concentration (including the additional highest sucrose concentration of 50%) did not vary significantly across the two consecutive exposures (*oil*: n_1,2_ = 109,98, χ^2^ = 2.42, p = 0.12; *geraniol*: n_1,2_ = 92,96, χ^2^ = 3.3 1, p = 0.07; *IPA*: n_1,2_ = 103,102, χ^2^ = 0.3, p = 0.6; *2H*: n_1,2_ = 106, 118, χ^2^ = 1.1, p = 0.3) (Fig. ESM1). These results confirm that the effect of pheromone components on sucrose responsiveness is robust and replicable.

### Effect of pheromone exposure on non-associative learning

In a second experiment, we determined whether pheromones also modulate experience-dependent behavior besides spontaneous reward responsiveness. We focused on habituation to antennal sucrose stimulation, which represents a case of non-associative learning^32–34^. Focus on this form of habituation was justified given its correlation with sucrose responsiveness^31^: bees highly responsive to sucrose display a lower degree of habituation and greater dishabituation than bees less responsive to sucrose. Based on these results, we expected geraniol and 2H to induce low and high habituation respectively; no effect of IPA on PER habituation was expected. In all cases, fifteen min after pheromone exposure (or mineral oil for control bees) we recorded PER occurrence during a series of thirty sucrose antennal stimulations with 10% sucrose solution delivered to the antennae and spaced by 10 s. Based on these responses, we computed for each bee a habituation score (number of sucrose stimulations eliciting PER), which ranged from 1 to 30.

**Figure 5:**
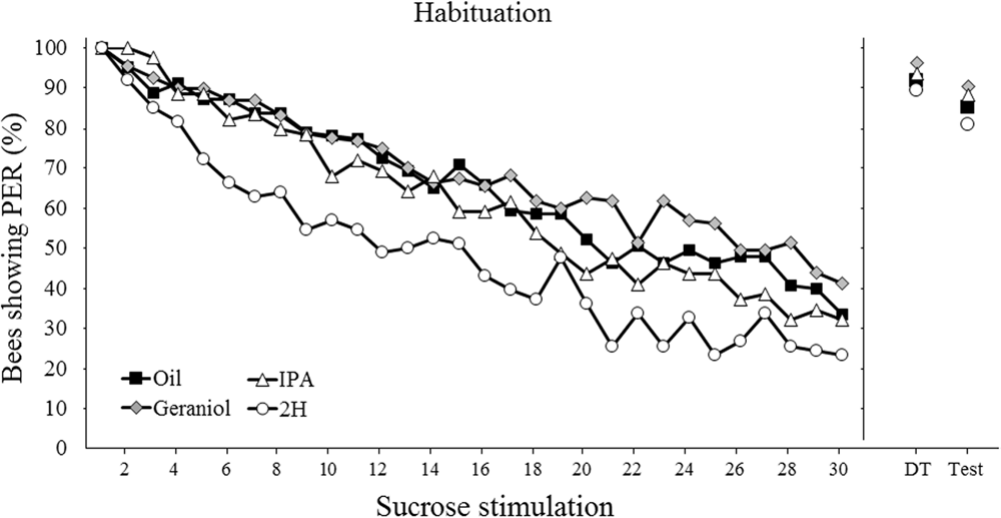
Pheromone exposure affects habituation to sucrose responses. Habituation curves of bees previously exposed to mineral oil (n = 123) or to the three pheromone treatments (geraniol: n = 107, IPA: n = 78, 2H: n = 86). Habituation was measured during 30 consecutive antennal stimulations with 10% sucrose solution. Ten seconds after the last habituation trial, bees were stimulated on the antennae with a 50% sucrose stimulation (Dishabituating Trial or DT) to induce dishabituation. Ten second after the DT, bees were stimulated with the original stimulus used during the training (i.e. 10% sucrose solution) to check for typical response recovery following dishabituation. 2H induced significantly more habituation than mineral oil (GLMM, n = 86, p = 0.0003). Neither geraniol nor IPA affected habituation (GLMM, *geraniol:* n = 107, p = 0.61; *IPA:* n = 78, p = 0.77). No significant differences in dishabituation according to treatments were observed. The DT as well as re-stimulating with the original dishabituating stimulus induced a significant response recovery, which did not differ between treatments. This recovery demonstrates that the observed decrease in PER to the 10% sucrose solution was a real case of habituation and was not due to sensory adaptation or fatigue.

**Figure 6:**
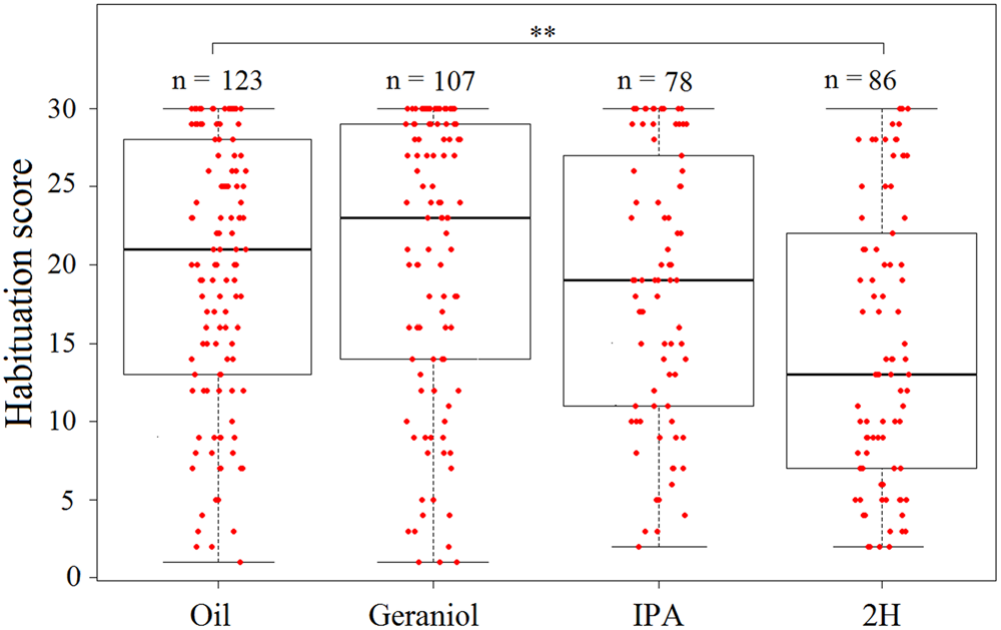
Pheromone exposure affects habituation to sucrose responses. Median, quartiles and max and min (upper and lower whiskers) SRS values of habituation scores (see Materials and Methods) for the groups exposed to mineral oil or to the different pheromone components (geraniol, IPA and 2H). Red dots represent individual bees. Bees with a score of 30 responded to all the 30 sucrose stimulations, i.e. did not show any habituation. *Non-responding bees* (score of 0, 8.2% of those exposed to oil, 13.0% to geraniol, 48.0% to IPA and 48.8% to 2H) were removed from the analysis as habituation cannot be assessed in this case. **p = 0.0003.

All groups exhibited habituation to antennal sucrose stimulation along trials as PER decreased significantly from the 1^st^ to the last habituation trial (GLMM, *trial*: χ^2^ = 1891, df = 1, p < 0.001) (Fig. 5). Yet, pheromone exposure affected the degree of PER habituation (GLMM, *pheromone:* χ^2^ = 25.42, df = 3, p < 0.001). Accordingly, the four groups of bees had different habituation scores (Kruskal-Wallis test, H = 25.25, df = 3, p < 0.001) (Fig. 6). In particular, bees exposed to 2H habituated faster (GLMM, n = 86, p = 0.0003) and exhibited higher habituation scores than bees exposed to mineral oil (Dunn’s test, *2H* *vs oil*: p = 0.0002). Neither geraniol nor IPA affected habituation speed (GLMM, *geraniol:* n = 107, p = 0.61; *IPA:* n = 78, p = 0.77) (Fig. 5) or habituation score (*geraniol vs oil*: p = 0.7; *IPA vs oil*: p = 0.7) (Fig. 6).

In order to evaluate whether the decrease of PER to successive stimulations with the 10% sucrose solution represents a true habituation phenomenon instead of being the result of fatigue and/or sensory adaptation, we assessed the effect of pheromone exposure on dishabituation (i.e. the fast recovery of a response that has undergone habituation, following a change in the parameters of the habituating stimulus^29, 30^). To this end, pheromone- and mineral-oil exposed bees that experienced the thirty antennal 10% sucrose solution stimulations received a single antennal 50% sucrose stimulation (dishabituation trial: DT) after the habituation procedure. Ten seconds later, the bees received a single stimulation (test phase) with the original stimulus used in the training (i.e. 10% sucrose solution) to check for typical response recovery following dishabituation.

In all groups, irrespective of the exposure treatment, PER increased after replacing the 10% habituation sucrose solution by the 50% sucrose solution (Fig. 5). A comparison between the response to the last habituation trial and that to the dishabituation trial (DT) showed a significant increase of PER in all cases (Wilcoxon test, *oil*: n = 123, Z = −8.37, p < 0.001; *geraniol*: n = 107, Z = −7.68, p < 0.001; *IPA*: n = 78, Z = −6.93, p < 0.001; *2H*: n = 86, Z = −7.55, p < 0.001). More importantly, the test with the original habituating stimulus (10% sucrose solution) after the DT showed in all cases a significant increase of PER compared to the response recorded in the last habituation trial (Wilcoxon test, *oil*: n = 123, Z = −8.00, p < 0.001; *geraniol*: n *=* 107, Z = −7.38, p < 0.001; *IPA*: n = 78, Z = −6.63, p < 0.001; *2H*: n = 86, Z = −0.07, p < 0.001, Fig. 5). These results demonstrated that the decreased number of responses observed over the habituation trials reflected true habituation and not fatigue or sensory adaptation. Indeed, the response recovery obtained both in the DT and when re-stimulating with the original dishabituating stimulus ruled out these possibilities and further arguments based on peripheral effects. Dishabituation responses to the original 10% sucrose solution stimulation after the DT did not differ between groups (*geraniol vs control:* χ^2^ = 1.50, df = 1, p = 0.22; *IPA vs control*: χ^2^ = 0.39, df = 1, p = 0.53; *2H* *vs control*: χ^2^ = 0.59, df = 1, p = 0.44), thus showing that pheromone exposure had no significant effect on this behavioral component.

## Discussion

Our study aimed at investigating the role of pheromones in behavioral plasticity in honey bees. To this end, we exposed bees to three pheromonal components of different valence and determined the effect of this exposure on an innate appetitive response and on non-associative learning. Response variations were measured fifteen min after pheromone exposure, i.e. when the pheromone components were no longer present, in order to assess response modulation rather than reflexive, acute responding to pheromone components. We focused on proboscis extension to sucrose stimulation as it allows assessing both innate sucrose responsiveness and experience-dependent changes in appetitive behavior in honey bees^25, 35^. After exposing bees to pheromone components that are typically encountered in different behavioral contexts such as foraging and nest defense, we observed significant changes in both sucrose responsiveness and habituation, a fact demonstrating that pheromones may modulate innate and experience-dependent responsiveness. Furthermore, we show that the direction of this modulation depends on the positive or negative valence of pheromones, i.e. on their signaling role in an appetitive or an aversive context.

Geraniol, a positive-valence pheromone component, which is used to mark profitable food sources, increased sucrose responsiveness (Figs 2 and 3). This result reflects an increase in appetitive motivation promoted by this pheromone component. Yet, geraniol had, unexpectedly, no effect on habituation to antennal sucrose stimulation (Figs 5 and 6). In this case, a resistance to habituation was expected, consistently with an enhanced appetitive motivation for sucrose. A possible explanation for this finding is that the concentration used as habituating stimulus (10%) was too high to appreciate the enhancing effect of geraniol. Indeed, the analysis of sucrose responsiveness showed that enhancing effects occurred for lower concentrations (0.1% and 0.3%) but not for all the other concentrations, including 10%.

IPA and 2H should decrease appetitive motivation by promoting alarm and aggressive responses to defend the nest. Such a decrease was indeed visible in the number of bees not responding to any sucrose concentration (Fig. 1: IPA: 40%; 2H: 60%), and in the decrease of sucrose responsiveness (Figs 2 and 3) and concomitant increase of habituation induced by 2H, but not by IPA (Figs 5 and 6). The high proportion of non-responding bees after exposure to 2H might be attributed to the reported anesthetic effect of this pheromone^36^. Yet, this effect is typically exerted on small hive enemies such as wax moth larvae (WML) and *Varroa* mites, which are paralyzed after a honey bee bite, but no report exists to our knowledge mentioning an anesthetic effect of 2H on other bees. Moreover, such an effect cannot explain the modulation of sucrose responsiveness and habituation by 2H, because our results were obtained after discarding all non-responsive individuals. Instead, the higher sensitivity of foragers to 2H may be explained considering that this substance, whose levels are higher in foragers than in guards^37^ has been suggested as a deterrent scent used to mark recently visited and depleted flowers in the appetitive context of food search^18^. Its negative valence could thus be transferred to the appetitive context provided by antennal sucrose stimulation. The case of IPA is different as this substance did neither affect sucrose responsiveness nor habituation. A similar effect was found by Urlacher *et al*.^38^ who reported the lack of difference in SRS between IPA-exposed bees and control bees exposed to mineral oil. It thus seems that IPA, despite of its signaling of noxious situations, may not be powerful enough to detract bees from their appetitive motivation. Although we still do not know the reasons for this lack of effect, it is worth noting that IPA also integrates floral fragrances^39^ of several species regularly visited by honey bees, and may thus constitute an appetitive signal when associated with sucrose stimulation. Previous research already showed that both IPA and 2H are repellent to honey bee foragers and able to stop immediately their foraging activity when encounter on flowers^21–23^. These innate responses occur with the concomitant presence of the releaser pheromones and are therefore spontaneous and reflexive.

The effect exerted by pheromones observed in the present study is in agreement with a model that has been recently proposed to explain the decision-making process underlying honey bee aggression^40^. In this model, bees would integrate a variety of different external and internal stimuli and factors to compute an overall “defensive score”. The threshold for aggression would thus be variable and dependent on multiple events. Among them, IPA presence would be crucial to set the threshold level, lowering it and facilitating aggression. This model accounts for the facts that IPA impairs appetitive learning in bees^38, 41^ and that some appetitive floral odors attenuate IPA-induced aggression in bees^40^. Our finding that pheromones with positive and negative valence directly affect sucrose responsiveness in an opposite way suggests that pheromones directly modulate thresholds of responsiveness, ultimately affecting decision making in bees.

Our study focused on three pheromone components but honey bees possess a much richer pheromonal repertoire with more than fifty pheromones acting in a variety of ecological contexts^9^. Other pheromones may act as further modulators of behavioral responsiveness. For instance, both the mandibular pheromone released by the queen and the pheromone produced by the brood induce a decrease in sucrose responsiveness^42^. Also, waggle-dancing bees release a hydrocarbon blend which promotes exit from the hive^43^, which could also be mediated by a change in sucrose responsiveness. In honey bees, sucrose responsiveness is tightly correlated with learning performance in appetitive conditioning^25, 31, 44–46^, thus accounting for the fact that the pheromones mentioned above also modulate learning performances^4, 38, 47^.

Overall, our findings indicate that pheromones can contribute to individual and colony level plasticity by modulating the bees’ motivational state and their learning performances. This conclusion introduces a novel perspective into the general appreciation of pheromone effects, usually considered restricted to the triggering of stereotyped responses, and it acknowledges the important role of pheromones for behavioral plasticity. Identifying the neural mechanisms underlying pheromone–induced plasticity may yield additional light into the question of how pheromones modulate behavior and orchestrate collective responses.

## Methods

### Animal preparation

Experiments were carried on from late April to the end of October 2015 using forager bees (*Apis mellifera ligustica*) caught at the entrance of several hives on the day of the experiment. Hives belonged to the experimental apiary of the Research Center on Animal Cognition, located in the campus of the University Paul Sabatier (Toulouse, France). Each day bees were randomly assigned to control and experimental groups and brought to the laboratory. Once in the laboratory, the bees were cold anaesthetized for 5 min and harnessed individually within a copper tube using adhesive tape placed in between the head and the thorax. Low-temperature melting wax was used to further immobilize the head such that bees could freely move only their antennae and mouthparts^48^. Proboscis extension response (PER) can be elicited in bees immobilized in this way by touching the antennae with sucrose solution. Once harnessed, the bees were fed with 5 μL of sucrose solution (50% w/w) to equalize the level of hunger across individuals and kept resting for 2Hours in a dark and humid place (~60%) at 25 ± 1 °C before proceeding with the experiment.

### Pheromone exposure

After the two-hour rest, harnessed bees were exposed either to mineral oil (control, n = 262) or to one of the three pheromone components: geraniol (n = 276), isopentyl acetate (IPA, n = 283) or 2-heptanone (2H, n = 147). All chemicals were purchased from Sigma-Aldrich (France). To this end, bees were individually enclosed in a 35 mL glass vial containing a 1 × 5 cm filter paper soaked with pheromone component (24% in mineral oil) or pure mineral oil as control (25 μL in each case) for 15 minutes. This amount of pheromone has been already used in a previous study^38^ and it would correspond to the natural situation of many bees scenting an attractive target, as it is typically the case. Fifteen minutes after exposure, bees were subjected either to the sucrose responsiveness assay or to the habituation assay (see below). After and before pheromone exposure, bees were allowed to drink water *ad libitum* in order to reduce the probability that they would respond to water in the sucrose responsiveness assay.

### Sucrose responsiveness assay

Sucrose responsiveness was quantified in harnessed bees by recording PER in response to increasing concentrations of sucrose, following a standard protocol^28, 44, 45^. Each bee was presented with six sucrose solutions of increasing concentration: 0.1%, 0.3%, 1%, 3%, 10%, and 30% (w/w), which were delivered to both antennae with the help of one toothpick^45^. Stimulations with distilled water delivered to the antennae were interspersed between successive sucrose stimulations to avoid sensitization due to sucrose. The inter-stimulus (either sucrose or water) interval was 2 minutes. Sucrose solutions were prepared using sucrose of analytical grade (Sigma-Aldrich, France) diluted in purified and deionized water (Milli-Q system, Millipore, Bedford, USA). Bees that did not respond to any sucrose concentration of the experimental series were presented with a 50% (w/w) sucrose solution at the end of the sequence and those not responding even to 50% sucrose were excluded from successive analyses^44^. We also discarded from the analysis bees responding to water to control for the effect of thirst on sucrose responsiveness^49^. The proportions of these bees were low (oil: 5.4%, geraniol: 1.0%, IPA: 3.2%, 2H: 2.6%). Moreover, bees exhibiting inconsistent responses to sucrose (i.e. responding to a lower but not to a higher sucrose concentration) were also discarded, as preconized by the standard method of sucrose responsiveness evaluation, because the lack of response to the higher concentration may be due to an uncontrolled motor problem and not to sucrose sensitivity itself. The proportions of these bees were again low in all treatments: 4.7% of those exposed to oil, 4.7% to geraniol, 3.6% to IPA and 0.7% to 2H. For each bee retained for the analysis, an individual sucrose response score (SRS) was calculated as the number of sucrose concentrations eliciting a PER (e.g., SRS = 3 for an individual responding to 3, 10, and 30% sucrose solution but not to lower concentrations). SRS ranged from 0 to 6. Bees with a SRS of 0 did not respond to any concentration (but responded to the additional sucrose concentration of 50% delivered at the end of the sequence) while bees with a SRS of 6 responded to all six sucrose concentrations.

To test whether the effect of pheromone exposure was consistent over time, the whole procedure (exposure and PER assessment to all sucrose concentrations) was repeated twice for each group of bees (N = 120 for each group). The two repetitions were spaced by two hours (from the first to the second exposure). Between successive tests harnessed bees were kept resting for one hour in a dark and humid place (~60%) at 25 ± 1 °C.

### Non-associative learning assay (habituation and dishabituation)

Harnessed bees were first assessed for their response to a 50% sucrose solution. Those that showed PER were then subjected to the 15 min exposure procedure described above (oil: n = 134, geraniol: n = 123, IPA: n = 150, 2H: n = 168) and, fifteen minutes later, trained following a habituation protocol. During training, harnessed bees were stimulated on both antennae 30 consecutive times with 10% sucrose solution for less than a second and an inter-stimulus interval of 10 seconds^31^. PER to antennal sucrose stimulation was quantified in each trial (yes/no). The dishabituation trial started 10 seconds after the last habituation trial and consisted of a single stimulation (dishabituation trial: DT) with a 50% sucrose solution delivered to both antennae. Ten seconds after the dishabituation trial, the bees received a test stimulation with the original stimulus used in the habituation phase (10% sucrose solution). In all cases PER to the stimulating solution was assessed.

Individual habituation scores were calculated as the number of stimulations eliciting a PER in the habituation phase and ranged, therefore, from 1 to 30. Bees that did not respond to the first sucrose stimulation in the habituation phase test were discarded from the analysis (8.2% of bees exposed to oil, 13.0% of those exposed to geraniol, 48.0% of those exposed to IPA and 48.8% of those exposed to 2H).

### Data analysis

Differences between the sucrose response score (SRS) of different groups of bees were analyzed using a Kruskal-Wallis test followed by post hoc pairwise comparisons based on a Mann-Whitney U test or Dunn’s test when appropriate. χ^2^ tests were used to compare the proportions of bees responding to different sucrose concentrations. For multiple comparisons, the alpha value was adjusted according to Holm-Bonferroni method. Sucrose responses (PER: 1 or 0) of individual bees in both the sucrose responsiveness and habituation/dishabituation assays were also examined using generalized linear mixed models (GLMMs) with a binomial error structure - logit-link function -, *glmer* function of R package *lme4* ^50^. For the sucrose responsiveness assay ‘response’ was entered as dependent variable, ‘pheromone’ as fixed factor and ‘sucrose concentration’ as covariate. ‘Individual’ identity (ID) was considered as a random factor in order to allow for repeated measurements. For the habituation/dishabituation assay, ‘response’ was the dependent variable, ‘pheromone’ was a fixed factor and ‘trial’ was entered as covariate. ‘Individual’ was considered as a random factor to account for repeated measures. For the dishabituation test, ‘habituation score’ of individual bees was entered in the model as a fixed factor. In all cases, we retained the significant model with the highest explanatory power (i.e. the lowest AIC value). The interaction pheromone * sucrose concentration was not significant in the full model and was, therefore, not included in the selected model for the sucrose responsiveness assay. It was, however, included in the selected model for habituation as in this case it was significant in the full model. We used Dunnett’s post-hoc tests to detect differences between the different groups (*glht* function from R package *multcomp* ^51^. All statistical analyses were performed with R 3.2.3 (R Development Core Team, 2016).

## Electronic supplementary material

Supplementary Information
