# Supplementary material for: Pheromones modulate reward responsiveness and non-associative learning in honey bees
Source: Sci Rep. 2017 Aug 29;7:9875. doi: 10.1038/s41598-017-10113-7 (PMC5574997; doi:10.1038/s41598-017-10113-7)
Supplement: Supplementary file 1 — Supplementary Information [file 41598_2017_10113_MOESM1_ESM.pdf]

# Supplementary Information

## Pheromones modulate reward responsiveness and non-associative learning in honey bees

David Baracchi, Jean-Marc Devaud, Patrizia d'Ettorre & Martin Giurfa

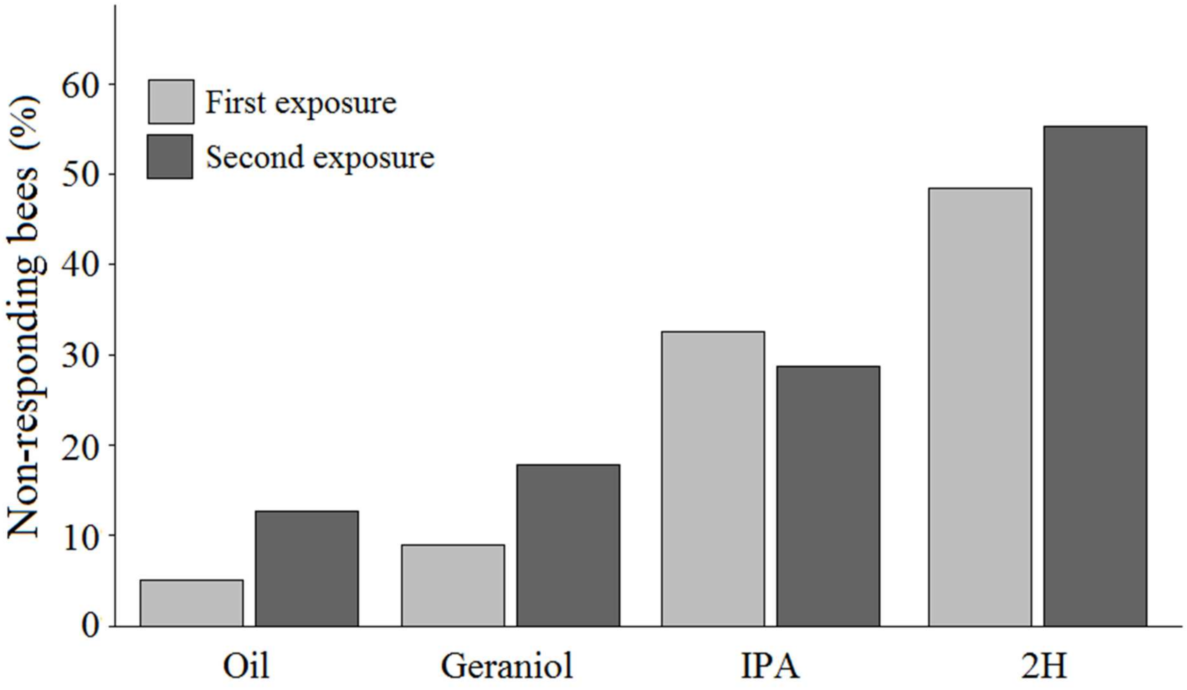


**Figure ESM1: Pheromone modulation of sucrose responsiveness remains invariable between repeated exposures.** Proportion of bees that failed to respond to any of the tested sucrose concentrations (including a 50% sucrose concentration) (*non-responding bees*) when evaluated after a first and second exposure to mineral oil (n = 120) or to one of the three pheromone components (geraniol, IPA and 2H; n = 120 for each group). In all groups, the proportion of *non-responding bees* was similar for the two successive exposures (*oil*: $\chi^2 = 2.42$, $p = 0.12$; *geraniol*: $\chi^2 = 3.3\ 1$, $p = 0.07$; *IPA*: $\chi^2 = 0.3$, $p = 0.6$; *2H*: $\chi^2 = 1.1$, $p = 0.3$).
